# Transient Enhanced GluA2 Expression in Young Hippocampal Neurons of a Fragile X Mouse Model

**DOI:** 10.3389/fnsyn.2020.588295

**Published:** 2020-12-03

**Authors:** Tue G. Banke, Andres Barria

**Affiliations:** Department of Physiology and Biophysics, University of Washington, Seattle, WA, United States

**Keywords:** fragile X mental retardation protein, glutamate receptor (AMPAR), LTP (long term potentiation), dendritic spines and memory, FMR 1 gene, NMDAR (NMDA receptor), circuit, synapses

## Abstract

AMPA-type glutamate receptors (AMPARs) are tetrameric ligand-gated channels made up of combinations of GluA1-4 subunits and play important roles in synaptic transmission and plasticity. Here, we have investigated the development of AMPAR-mediated synaptic transmission in the hippocampus of the Fmr1 knock-out (KO) mouse, a widely used model of Fragile X syndrome (FXS). FXS is the leading monogenic cause of intellectual disability and autism spectrum disorders (ASD) and it is considered a neurodevelopmental disorder. For that reason, we investigated synaptic properties and dendritic development in animals from an early stage when synapses are starting to form up to adulthood. We found that hippocampal CA1 pyramidal neurons in the Fmr1-KO mouse exhibit a higher AMPAR-NMDAR ratio early in development but reverses to normal values after P13. This increase was accompanied by a larger presence of the GluA2-subunit in synaptic AMPARs that will lead to altered Ca^2+^ permeability of AMPARs that could have a profound impact upon neural circuits, learning, and diseases. Following this, we found that young KO animals lack Long-term potentiation (LTP), a well-understood model of synaptic plasticity necessary for proper development of circuits, and exhibit an increased frequency of spontaneous miniature excitatory postsynaptic currents, a measure of synaptic density. Furthermore, *post hoc* morphological analysis of recorded neurons revealed altered dendritic branching in the KO group. Interestingly, all these anomalies are transitory and revert to normal values in older animals. Our data suggest that loss of FMRP during early development leads to temporary upregulation of the GluA2 subunit and this impacts synaptic plasticity and altering morphological dendritic branching.

## Introduction

Loss of the fragile X mental retardation protein (FMRP) in the brain causes the fragile X syndrome (FXS), a leading monogenic cause of often severe intellectual disability which is characterized by moderate-to-severe mental retardation. FXS is the most common inherited intellectual disability syndrome (Santoro et al., 2012) and is considered as the most common single-gene condition associated with autism spectrum disorder (ASD; Hernandez et al., 2009). FMRP is highly expressed throughout the brain, including at synapses, where it may play critical roles regulating dendritic properties, synaptogenesis, and synaptic function. FMRP is encoded by the *Fmr1* gene that contains a CGG trinucleotide repeat, that, when expanded can results in abnormal DNA hypermethylation and transcriptional silencing of FMRP (Penagarikano et al., 2007; Garber et al., 2008). FMRP is involved in mRNA regulation of multiple downstream targets including members of the glutamate receptor family, thereby impacting the normal development of neurons, synapses, and brain circuits (Contractor et al., 2015). Furthermore, recent studies suggest that FMRP can directly regulate the intrinsic properties of neurons *via* direct interactions with potassium (Kv) and HCN channels in a cell-type-specific manner (Kalmbach et al., 2015).

Loss of FMRP results in abnormal neuronal structure and function in a brain-region and cell-type-specific manner, producing complex effects on circuit function in different sensory systems (Comery et al., 1997) and varied, sometimes contradictory, effects on synaptic properties and developmental plasticity (McBain and Fisahn, 2001; Yang et al., 2014).

The release of the neurotransmitter glutamate from presynaptic vesicles activates postsynaptic glutamate-gated ion channels including α-amino-3-hydroxy-5-methyl-4-isoxazolepropionic acid receptors (AMPARs). AMPA receptors play critical roles in synaptic signaling and plasticity, and AMPAR dysfunction is implicated in a variety of nervous system disorders (Bowie, 2008; Salpietro et al., 2019). Long-term potentiation (LTP) and long-term depression (LTD) are two well accepted and understood cellular models of synaptic plasticity (Lüscher and Malenka, 2012) that increase or decrease synaptic strength respectively. Previous work indicates an enhanced LTD in adult FMRP knock-out (KO) mice (Huber et al., 2002; Toft et al., 2016), however, this seems to be an age-dependent phenomenon as younger animals (<p21) did not show a difference in the amount of LTD induced (Toft et al., 2016). Discrepancies on whether LTP is affected in FMRP KO animals also exist and while it has been reported to be impaired in FMRP KO animals (Hu et al., 2008), it seems to be increased in a different FXS animal model that uses a double genetic manipulation to completely knock out the protein (Pilpel et al., 2009). The reasons for this discrepancy could be differences in the knockout model, age of the animals used, and/or differences in the LTP induction protocols used.

The development of excitatory synapses happens in a very tightly orchestrated manner; they are built as a complex of scaffolding proteins that link signaling proteins, cell adhesion molecules, and members of the glutamate receptor family to the microfilament-based cytoskeleton in dendritic spines. FMRP has been found to associate with the mRNA of various synaptic proteins including ionotropic glutamate receptor (iGluRs; Schütt et al., 2009; Edbauer et al., 2010), which could indicate that altered expression of iGluRs is involved in the pathophysiology of FXS.

Little is known about the role of FMRP in the early development of synapses and neuronal properties. A better understanding of the developmental profile of glutamatergic synaptic properties in the absence of FMRP is necessary to better understand how synaptic plasticity and the normal development of synapses are affected in FXS. Here, we studied the development of glutamatergic synapses, their synaptic plasticity properties, and the development of dendritic branching in mice hippocampal CA1 pyramidal neurons at different developmental stages from postnatal 6–33.

## Materials and Methods

### Animals

We used male *Fmr1* KO mice (FMRP KO, RRID:IMSR_JAX:003025, The Jackson Laboratory), and the C57BL/6J WT mice for this study. The *Fmr1* KO mouse was bred in the C57BL/6J background in a non-littermate fashion. All animals were kept on a 12:12 h light:dark cycle with a constant room temperature and provided with *ad libitum* food and water. The animal experiments were conducted following the animal care guidelines of the University of Washington under approved IACUC protocols.

### Slice Preparation

The animals were decapitated and the brains were rapidly removed and placed in ice-cold “slicing solution” equilibrated (130 NaCl, 3.5 KCl, 1.25 NaH_2_PO_4_, 24 NaHCO_3_, 10 D-Glucose, 0.5 CaCl_2_, and 5 MgCl_2_) with 95% O_2_/5% CO_2_ mixture, pH 7.4 and 300 μm coronal slices were prepared. In LTP experiments, CA3 was surgically removed immediately after sectioning. After cutting, the slices recovered at 32°C for 1 h before leaving at RT.

### Stimulation/Recording

Individual slices were placed in a submerged tissue slice chamber, where the temperature was maintained at 28.0 ± 1°C. Slices were perfused with carbogenated artificial cerebrospinal fluid (ACSF) with a flow rate through the chamber of 2.5 ml/min. ACSF was exactly like slicing solution except Mg^2+^ was lowered to 1.5 mM and Ca^2+^ was raised to 2 mM. A stimulating electrode was placed on the surface of the Stratum radiatum of area CA1 to stimulate the Schaffer collateral/commissural fibers. For LTP experiments, a second stimulating electrode was placed in Stratum radiatum near the subiculum and used to evoke responses in a control pathway that did not receive pattern stimulation. LTP was induced by stimulating the axons at 3 Hz for 120 s while clamping the cell at 0 mV.

Whole-cell voltage-clamp experiments were recorded from CA1 pyramidal neurons using the following intracellular solution (in mM): 115 CeMeSO_4_, 20 CsCl, 10 Hepes, 2.5 MgCl_2_, 4 Na_2_ATP, 0.4 Na3GTP, 10 NaPhosphatase, 0.6 EGTA, 100 μm spermidine, 5 μM QX314; pH was set at ~7.4 with CsOH; osmolarity was set at ~290. For all recordings except LTP recordings, cells were allowed to rest for ~5 min after whole-cell configuration was established. Uncompensated series resistance (Rs) was monitored continuously throughout recordings with −5 mV voltage steps before stimulus steps.

Also, either 100 μM picrotoxin or 20 μM bicuculine and 3 μM CGP55845 were routinely added to block GABA_A_ receptors and GABA_B_ receptors mediated transmission, respectively. For miniEPSCs, tetrodotoxin (TTX, 1 μM) and DL-2-Amino-5-phosphonopentanoic acid (APV; 100 μM) was added to block sodium channels and NMDARs, respectively.

Other antagonists and blockers used: 2,3-dihydroxy-6-nitro-7-sulfamoyl-benzo[f]quinoxaline [NBQX (10 μM)], and philanthotoxin-74 [PhTx (5 μM)]. All chemicals used were acquired from either Tocris or Sigma–Aldrich.

### *Post hoc* Anatomical Reconstruction and Imaging

For morphological reconstruction, in a subset of whole-cell recordings, 0.1% neurobiotin (Vector labs) were added to the internal solution just before the experiment. Slices were fixed over-night in 3% glutaraldehyde at 4°C, permeabilized in 0.1% Triton X-100, and incubated with Alexa 488-conjugated avidin. Slices were mounted on gelatin-coated slides using Mowiol mounting medium (Sigma) and imaged using a Zeiss LSM 710 confocal microscope (LSM 710). A 20× dry or 63× oil immersion objective was used for acquiring images of neurons. Image size for dendrite analysis was set at 425 × 425 μm and image size for spine analysis was set at 135 × 135 μm, respectively. Images were scanned with an interval of 0.5 μm along the Z-axis.

The maximum projection of Z-stacks was obtained using ImageJ for the spine and dendritic analysis. ImageJ’s “Simple Neurite Tracer” (NIH) was used for dendritic reconstruction and Sholl analysis. Secondary apical branches (each dendritic length of 50–200 μm) 50–120 μm from the soma were manually analyzed for spine density and morphology using NeuronStudio (Computational Neurobiology and Imaging Center at the Neuroscience Department of the Mount Sinai School of Medicine, New York, NY, USA). One dendritic section per neuron and 1–3 neurons per animal was analyzed. A total of 17-21KO and 23–27 WT neurons were analyzed for the spine and dendritic morphology in the P6–9 and P14–19 groups, respectively.

The length of the spines was calculated as the difference from the dendritic surface to the tip of the head.

All statistical analysis was performed using GraphPad Prism 6 (GraphPad Software Inc.) or Microsoft Excel. For imaging, dendritic and analysis, and mEPSC analysis, the investigator was blinded with regards to the genotype and age of the animals. Each mEPSC was manually selected offline (MiniAnalysis; Synaptosoft, Decatur, GA, USA).

Significant differences between groups were tested using unpaired Student’s *t*-tests, Kolmogorov–Smirnov (K–S), or one-way ANOVA (Tukey’s) when appropriate. Asterisk on figures indicates statistical significance (*p < 0.05; **p < 0.01; ***p < 0.001).

## Results

Altered synaptic structure and function is a well-known hallmark of FXS and thus, here we wanted to compare early synaptic development of glutamate receptors with a focus on AMPA receptors in FXS and WT hippocampal slices.

### AMPA-NMDAR Ratio

**Figure 1:**
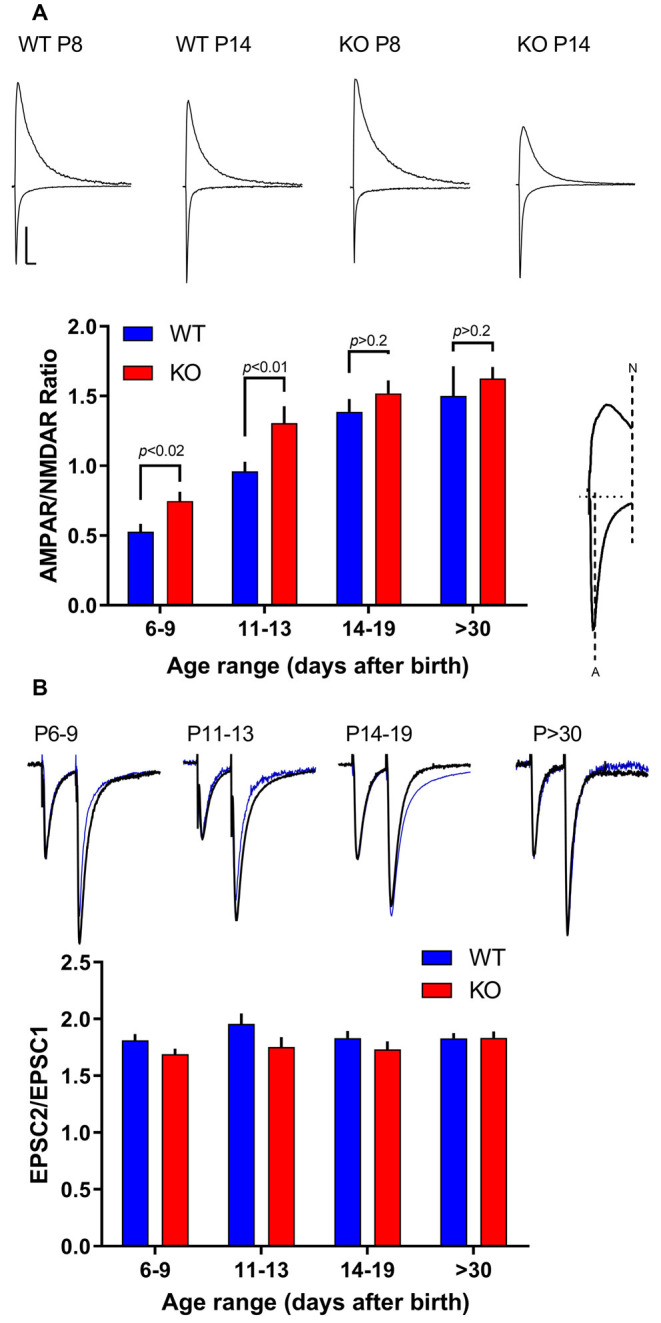
Amplitude of AMPA-type glutamate receptor (AMPAR) and NMDAR-mediated evoked postsynaptic currents (EPSCs) during development. **(A)** Developmental profile of the AMPA Receptor to NMDA Receptor ratio. Top, sample traces of evoked responses from CA1 pyramidal cells recorded in voltage-clamp at −60 mV or +40 mV from wild type (WT) and fragile X mental retardation protein (FMRP) knock-out (KO) slices as indicated. Scale bar = 50 ms and 50 pA. Bottom, population data of AMPAR to NMDAR ratio from WT neurons (blue bars) from slices P6–9 (*n* = 17), P11–13 (*n* = 42), P14–19 (*n* = 47), and *P* > 30 (*n* = 23). Red bars are from FMRP KO neurons from slices at same age groups (P6–9: *n* = 36; P11–13: *n* = 16; P14–19: *n* = 37; *P* > 30: *n* = 24). The amplitude of the AMPAR-mediated response was measured at the pick of the response at −60 mV. The amplitude of the NMDAR response was measured at +40 mV 80 ms after the stimulus artifact as indicated in the example on the right. Student’s *t*-test *p*-values are indicated in the figure. **(B)** Paired-pulse facilitation (PPR) in FMRP KO and WT CA1 neurons at different ages as indicated. Top, superimposed sample traces of evoked responses from CA1 pyramidal cells recorded in voltage-clamp at −60 mV from WT (thick line) and FMRP KO slices (blue line) as indicated. The traces were normalized to the first peak. PPR quantified as shown in the bar graph below, with the two stimuli delivered 50 ms apart (recorded at −60 mV). n values for WT neurons from P6–9 to *P* > 30 are: 10, 31, 36, 12. *n*-values for KO neurons from P6–9 to *P* > 30 are: 26, 10, 25, 14. No significant difference was found between WT and KO within age groups (*p* > 0.05).

First, to investigate glutamatergic neurotransmission in hippocampal synapses of FXS animals during postnatal development, we compared the amplitude of evoked postsynaptic currents (EPSCs) mediated by AMPARs and NMDARs in acute mice hippocampal slices at different developmental stages from early development (P6) to young adult (P33). In WT slices, an increase in the AMPAR to NMDAR ratio was observed from 0.53 ± 0.06 at P6–9 to 1.50 ± 0.21 at *P* > 30 (Figure 1), likely reflecting a shift from more silent synapses, i.e., synapses containing only NMDARs, to synapses with a larger content of AMPARs in the developing hippocampus (Liao et al., 1995). Overall, in KO slices the same picture emerged: a shift over time in the AMPAR/NMDAR ratio from 0.75 ± 0.07 at P6–9 to 1.63 ± 0.08 at *P* > 30.

Here, we report no significant difference in the AMPAR/NMDAR ratio between WT and KO in any group tested older than P14. However, in younger age groups (P6-9 and P11-13) a significant increase in the AMPAR/NMDAR ratio was observed in the KO (*p* < 0.02), suggesting a larger AMPAR component or a smaller NMDAR component in KO slices (Figure 1A). In the same recordings, in agreement with several other labs (Pfeiffer and Huber, 2007; Pilpel et al., 2009; Zhang et al., 2009; Deng et al., 2011; Eadie et al., 2012), no significant change in pair-pulse ratio was observed (Figure 1B) implying no changes in the probability of neurotransmitter release.

Due to changes in stimulation configuration across slices, it is not pertinent to compare absolute values of AMPAR or NMDAR responses between KO and WT slices. However, we noticed that in KO slices younger than P14 AMPAR responses were consistently larger than in their WT slices from P6–9 WT animals had AMPAR responses of −31 ± 3 pA (*n* = 17) while slices of the same age from KO animals have an average response of −55 ± 5 pA (*n* = 41; *p* < 0.01 Student’s *t*-test; data not shown). Slices from P11–13 animals also showed a significant difference with average amplitudes of −40 ± 3 pA in the WT slices (*n* = 42) and −57 ± 7 pA in KO slices (*n* = 16; *p* < 0.05; data not shown). AMPAR responses in slices from animals older than P14 were not significantly different. This hinted that AMPAR mediated transmission in KO mice could be altered early in development. Next, we decided to focus on the development of AMPAR mediated transmission in FMRP KO slices to test whether an abnormal AMPAR-mediated transmission occurs early in development.

### GluA2 Is Altered in the KO During a “Critical Maturation Period”

The AMPAR subunit GluA2 dictates important biophysical properties like Ca^2+^ permeability through the channel pore allowing it to play important roles in many Ca^2+^-dependent cell processes downstream of channel activation like synaptic plasticity. AMPARs lacking GluA2 subunits have high Ca^2+^ permeability, often called Ca^2+^-permeable AMPARs (CP-AMPARs), and exhibit inward rectification caused by intracellular polyamine block. In contrast, AMPARs containing GluA2 have low calcium permeability, called calcium impermeable-AMPARs (CI-AMPARs), and exhibit a linear current-voltage (I–V) relationship. Thus, the shape of the AMPAR I–V curve reveals whether GluA2 is present or not in the AMPAR complex (Isaac et al., 2007). We define a rectification index (RI) calculated as the amplitude of AMPAR-mediated currents recorded in voltage-clamp while holding the cell at +40 mV over the amplitude of AMPAR-mediated currents recorded at a holding potential of −60 mV. The presence of GluA2 would mean a linear I/V curve, therefore an RI closer to 0.67 (=40/60).

**Figure 2:**
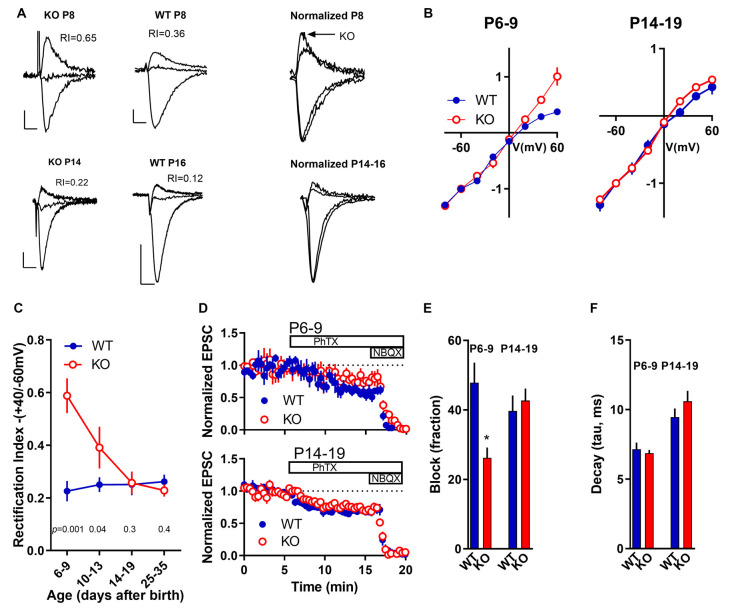
Developmental subunit composition of synaptic AMPARs in the FMRP KO. **(A)** Sample traces of AMPAR EPSCs recorded at −60, 0, and +40 mV in the presence of 100 μM DL-2-Amino-5-phosphonopentanoic acid (APV) in CA1 neurons from FMRP KO or WT neurons as indicated. To the right are the same traces with amplitude normalized to −60 mV. Scale bar = 10 ms and 50 pA. **(B)** Normalized current-voltage plot from P6–9 and P14–19 neurons from KO (white dots; *n* = 13–22) and WT slices (black dots; *n* = 8–25). **(C)** Rectification Index (RI) was calculated as the ratio of responses at +40 mV and −60 mV for KO neurons (white dots) and WT neurons (black dots). *n* values for WT neurons from P6–9 to *P* > 30 are: 16, 17, 9, 5. *n* values for KO neurons from P6–9 to *P* > 30 are: 10, 11, 17, 10. Student’s *t*-test *p*-value comparing KO vs. WT at each specific age is indicated in the figure. **(D)** Blockade of AMPAR-mediated currents with PhTx-74. After a 5 min baseline, PhTx was applied to the perfusion bath. Then, 20 μM of the general AMPAR blocker NBQX was added to the bath. Effect of PhTx in slices P6–9 (top) and in P14–19 (bottom) in WT neurons (black dots; *n* = 6–8) and KO neurons (white dots; *n* = 7–8). **(E)** The fraction of PhTx-74 blockade measured at the 15–18 min time window in slices as indicated. The asterisk indicates *p* < 0.05 significant difference between P6–9 WT and KO. **(F)** The decay phase of AMPAR-mediated responses was fitted with a single exponential curve and the time constant (tau) was estimated. No significant differences between WT and KO neurons at each age group were observed.

WT slices exhibit an overall rectification value of 0.28 ± 0.12 (*n* = 59) that remains constant through the periods studied (Figures 2A–C), suggesting the presence at synapses of mostly GluA2 lacking AMPARs. However, in slices from KO animals, early in development (<P14), we found a higher RI index (0.39–0.59; Figures 2A–C) indicating an enhanced synaptic content of GluA2-containing AMPARs. Because GluA2-containing AMPARs are Ca^2+^ impermeable, this could have important consequences in the normal development of synapses, development of dendrites and spines, and other Ca^2+^-dependent processes like synaptic plasticity.

This relatively enhanced synaptic GluA2-containing AMPARs seems to go back to normal in slices from KO animals older than P14, as indicated by an RI similar to WT slices. This data suggests that overexpression of GluA2 early in development is only temporary.

This enhanced GluA2 containing AMPARs could indicate the insertion of a completely “novel” AMPAR population, like those composed of the GluA4 and GluA2 subunits to synapses already containing GluA1/GluA2 receptors. The expression of GluA4 is known to be restricted to the first postnatal week in Hippocampus (Zhu et al., 2000) and thus is an interesting candidate. This could be detected in the kinetics of decay of the AMPAR-mediated EPSCS because of the fast kinetics of Glu4 subunits (Lomeli et al., 1994). However, we detect no difference in the decay times of EPSCs in young slices (P6-9; Figure 2F) arguing against such a scenario.

To study this phenomenon in more detail, we took advantage of philanthotoxins (PhTXs). AMPARs are blocked by PhTXs which are polyamine toxins isolated from the venom of wasps and spiders (Strømgaard et al., 2005). However, the degree of the block depends upon the AMPAR subunit composition. Thus, AMPARs lacking the GluA2 subunit have a higher affinity towards PhTXs that AMPAR containing GluA2 subunits. Thus, we tested the PhTX analog PhTX-74 (Poulsen et al., 2013) on young (P6-9) vs. older slices (P14-19). In slices from young animals (P6-9), after establishing a 5-min baseline, the application of PhTx-74 blocked a larger fraction of AMPAR-mediated EPSCs in WT slices than in KO slices (*p* < 0.05; Figure 2D). In slices from older animals (P14-19), no difference in the amount of blockade of AMPAR responses was observed between WT and KO slices (*p* > 0.05; Figures 2D,E). This experiment confirms that in the younger FXS Hippocampus, while both GluA2-containing and GluA2-lacking AMPARs exist, a larger proportion of the AMPARs contain the GluA2 subunit in KO than in WT.

### Lack of LTP at Synapses in Young KO Animals

**Figure 3:**
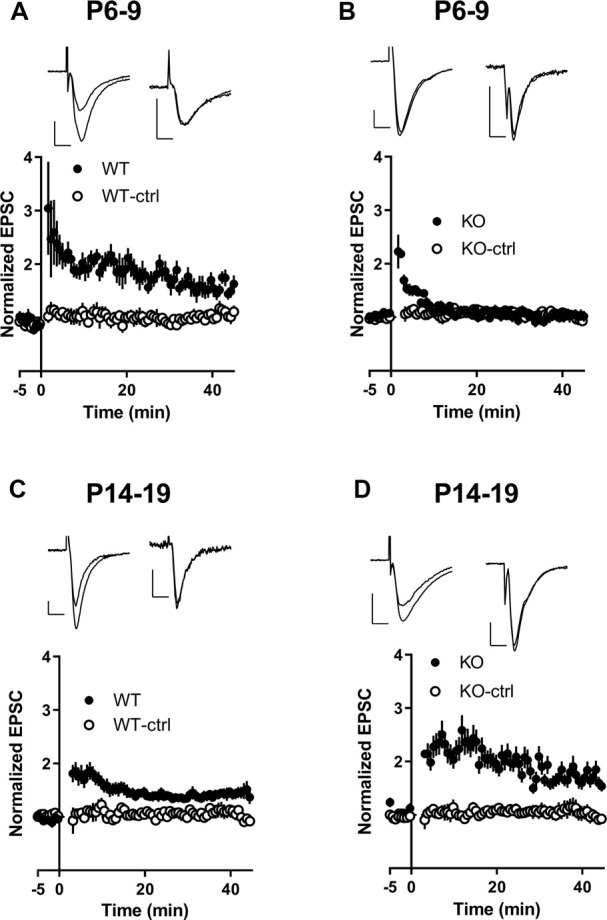
Long-term potentiation (LTP) of Schaffer collateral-CA1 synapses in FMRP KO during early development. **(A–D)** In the presence of a GABAA inhibitor, after obtaining a 5-min baseline, LTP was induced in CA3-CA1 synapses by stimulation of Schaffer collaterals by stimulating the axons at 3 Hz for 2 min while clamping the cell at 0 mV (black dots). A second stimulation electrode was used as a control (white dots). This protocol readily induced LTP in WT P6–9 slices (**A**; *n* = 8) and P14–19 slices (**C**; *n* = 13). **(B,D)** LTP induction in KO slices P6–9 (**B**; *n* = 12) and KO slices P14–19 (**D**; *n* = 7). Insets are examples of evoked EPSCs from the baseline period and 40 min after LTP induction. The left pair of traces are from the test path and the right are from the control path. Scale bars 10 ms and 50 pA.

AMPARs play important roles in the initiation and maintenance of synaptic plasticity, including LTP (Malinow et al., 2000). Since we observed an increase in GluA2-containing AMPARs in KO animals in an age-depending manner we next attempted to induce LTP in pyramidal neurons using a standard paired stimulation protocol (see “Materials and Methods” section). Robust LTP was obtained in slices from young (P6-9) and older (P14–19) WT animals as described before (Yasuda et al., 2003; Palmer et al., 2004). In slices from KO animals, LTP was induced normally in slices from older animals (P14–19) but it failed to be induced in slices from the younger age group (P6–9; Figure 3).

Next, we analyzed the development of synaptic connections by recording miniature EPSCs (mEPSCs) in our slices. We used the mEPSC inter-event interval (IEI) as an indicator of functional synapse number (Gambrill and Barria, 2011), and mEPSC amplitude as a measurement of individual synaptic strength.

**Figure 4:**
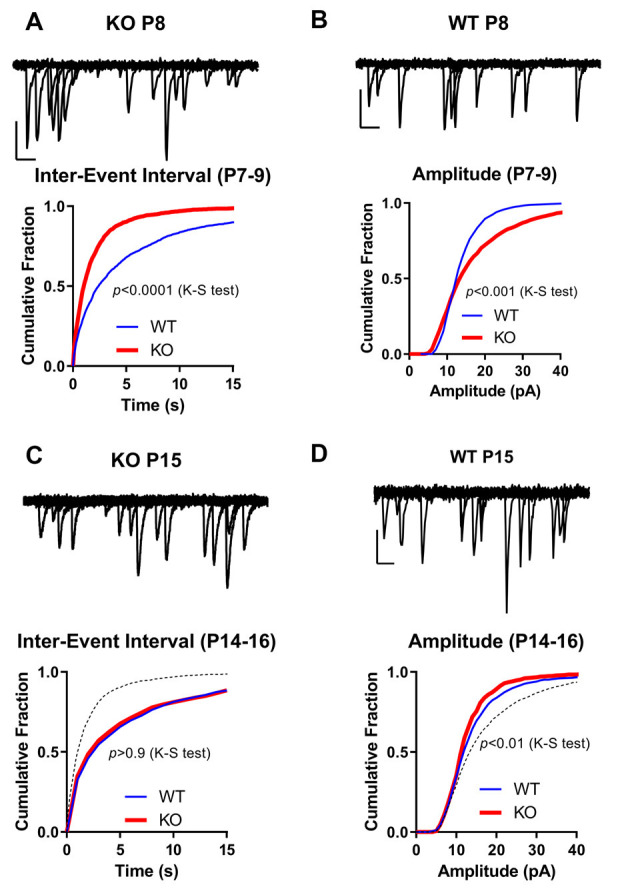
Spontaneous miniature EPSCs in FMRP KO slices. **(A–D)** Cumulative distribution of Inter-Event Intervals **(A,C)** of spontaneous events in CA1 neurons recorded at −60 mV in the presence of TTX and the amplitude of those events **(B,D)** from WT (blue line; *n* = 19–29) or FMRP KO slices (*n* = 31–31). **(A,B)** P6–9. **(C,D)** P14–16. Kolmogorov-Smirnov (K-S) test *p*-value is shown in the figure. For comparison, superimposed on Figure C is WT P6-9 (dotted black line; from **A**). Note the large increase in frequency of mEPSCs (decrease in Inter-Event Intervals) and in the amplitude of those events in KO neurons from slices P6-9. On top of each graph are shown 10 superimposed 250 ms long mEPSC events of KO P8 **(A)**, WT P8 **(B)**, KO P15 **(C)**, and WT P15 **(D)**. Scale bars: X-axis: 20 ms; Y-axis: 20 pA.

The mEPSCs were recorded in the presence of TTX and APV to block sodium channels and NMDARs, respectively. In the P14–19 age group, mEPSC IEI in KO was not significantly different from WT (Table 1), whereas the younger KO group (P6–9) exhibited increased mEPSC IEI compared to both the WT (P6–9) group and the P14–19 age groups (Table 1; Figures 4A–D).

**Table 1:** mEPSC statistics.

	IEI (ms)	Amp (pA)	Rise (ms)	Decay (ms)	*n*
**P6–9**					
*p*	0.03	<0.001	0.21	0.19	
Average WT	2.9 ± 0.1	10.5 ± 0.5	2.7 ± 0.2	3.8 ± 0.3	25
Average KO	1.1 ± 0.1	16.8 ± 1.6	2.3 ± 0.1	3.2 ± 0.2	26
**P14–19**					
*p*	0.79	0.32	0.054	0.6	
Average WT	3.6 ± 0.1	15.2 ± 1.1	2.3 ± 0.06	3.8 ± 0.2	21
Average KO	3.2 ± 0.1	13.5 ± 1.1	2.5 ± 0.1	4.0 ± 0.3	21

*Recordings of miniature excitatory postsynaptic currents (mEPSCs) from P6–9 (top) or P14–19 (bottom) CA1 pyramidal neurons in the presence of tetrodotoxin (TTX) and DL-2-Amino-5-phosphonopentanoic acid (APV). Data are presented as average ± SEM. Inter-event interval (IEI), amplitude (Amp), rise time, decay time, and number (n) of recordings are shown. *p* calculated from Student’s *t*-test are shown at the top*.

The amplitude of mEPSCs was increased in slices from younger KO animals when compared to WT slices in the same age group. On average, the amplitude of mEPSCs in KO slices at the P6–9 age group was 17 ± 2 pA, significantly higher than their WT counterpart of 10 ± 1 pA (Table 1; *p* < 0.01; Student’s *t*-test; Figures 4A–D). The amplitude in slices from older animals was not significantly different between KO animals and WT; 15 ± 1 pA in WT and 14 ± 1 pA in KO (*p* > 0.05; *t*-test). As shown in Table 1 there was no significant difference in either rise time or decay time of mEPSCs among the groups.

This data suggests that in animals lacking FMRP, an early increase in synaptic connections exist as indicated by an increase in mEPSCs. Interestingly, this apparent increase in the number of synapses seems to go back to normal as the mEPSC frequency is not different later. More importantly, an increase in the amplitude of mEPSCs is observed in these early synapses, suggesting they may be already be potentiated, thus occluding induction of LTP as shown in Figure 3.

### Dendritic Morphology

Synaptic plasticity, in particular LTP, it is an important mechanism to stabilize synapses and proper dendritic branching. Considering that early in development, FMRP KO animals show a deficit in LTP we decided to investigate whether this affected the normal dendritic branching of apical and basal dendrites in CA1 pyramidal neurons.

**Figure 5:**
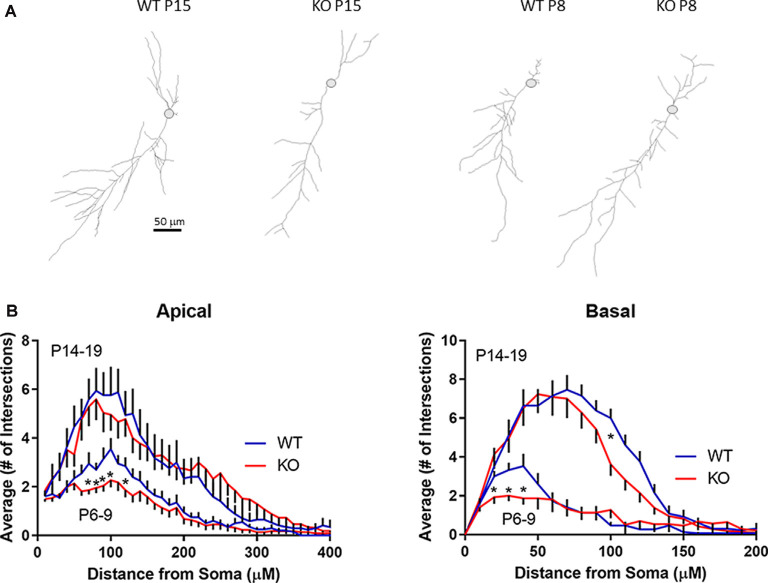
Development of dendritic branching in FMRP KO CA1 neurons. **(A)** Examples of skeleton drawings of CA1 neurons from slices as indicated. Neurons were backfilled with biotin for *post hoc* morphological analysis. Scale bar = 50 μm. **(B)** Sholl analysis indicating the average number of intersections of apical dendrites (left) or basal dendrites (right) with concentric circles spaced 10 μm apart from P6–9 or P14–19 pyramidal neurons from either FMRP KO slices (red line; *n* = 23 and *n* = 27, respectively) or WT slices (blue line; *n* = 21 and *n* = 17, respectively). Asterisks indicate *p* < 0.05 *t*-test statistical significance when comparing WT and KO slices within a particular age group.

**Figure 6:**
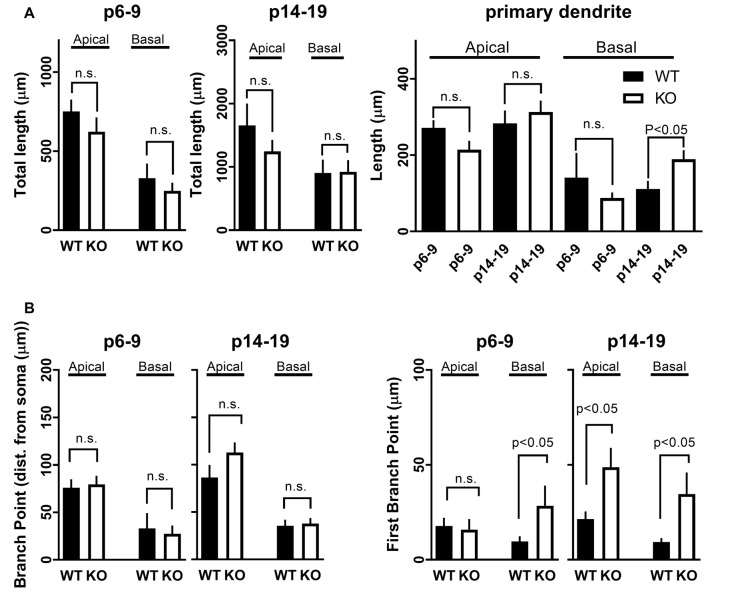
Development of dendritic branches in CA1 pyramidal neurons in FMRP KO mice. **(A)** Total length of dendritic branches in CA1 neurons either WT (black bars) or FMRP KO (white bars). The length of apical or basal dendrites was measured in slices either P6–9 (left) or slice P14–19 (right). The Student’s *t*-test shows no significance between WT and FMRP KO neurons within the different age groups or types of the dendrite. (Right) Length of dendritic branches broken down into primary dendrite from CA1 neurons as indicated. **(B)** Distance from soma to average all (left) or first (right) branching point in CA1 neurons from either WT (black bars) or FMRP KO (white bars) from slices P6–9 or slices P14–19 as indicated in Figure. The Student’s *t*-test shows significance (*p* < 0.05) between WT and FMRP KO neurons within the different age groups for the first branch point. n.s.: not significant.

Recording pipettes were backfilled with neurobiotin for *post hoc* neuronal reconstruction. Each reconstructed neuron was built from a series of dendritic branch fragments, each attached to the primary dendrite at specific branch points (Figure 5A). Although we found no significant difference in the total number of dendritic fragments within each age group (data not shown), no significant change in overall apical or basal total dendritic length (Figure 6A), and no significant difference in the average branch points, we did find a significant difference in the first dendritic branch point between KO and WT. Thus, the first branch point was located further away from the soma in KO in both the basal and apical dendritic tree (Figure 6B). In support of this, Sholl analysis did reveal similar significant differences in the number of intersections between KO and WT neurons within the first ~100 μm from the soma in both apical and basal dendritic tree (Figure 5B). Combined, these data suggest that WT neurons have, on average, a slightly denser dendritic tree structure near the soma than KO neurons.

Finally, a prominent neuronal phenotype described, and frequently cited, in the FMRP KO mouse as well as in FXS patients is an excessive proportion of thin immature-like tortuous spines. It is important to point out that this phenotype corresponds to cerebral cortex neurons of adult (16-week-old) mice (Comery et al., 1997). Given the age-dependent alterations in synaptic GluA2 expression, lack of hippocampal LTP in young animals, and a less dense dendritic tree in young FMRP KO animals, we next decided to test also dendritic morphology at this critical early stage of development. Secondary apical branches 50–120 μm from the soma of CA1 pyramidal neurons were selected and analyzed for spine density and morphology.

**Figure 7:**
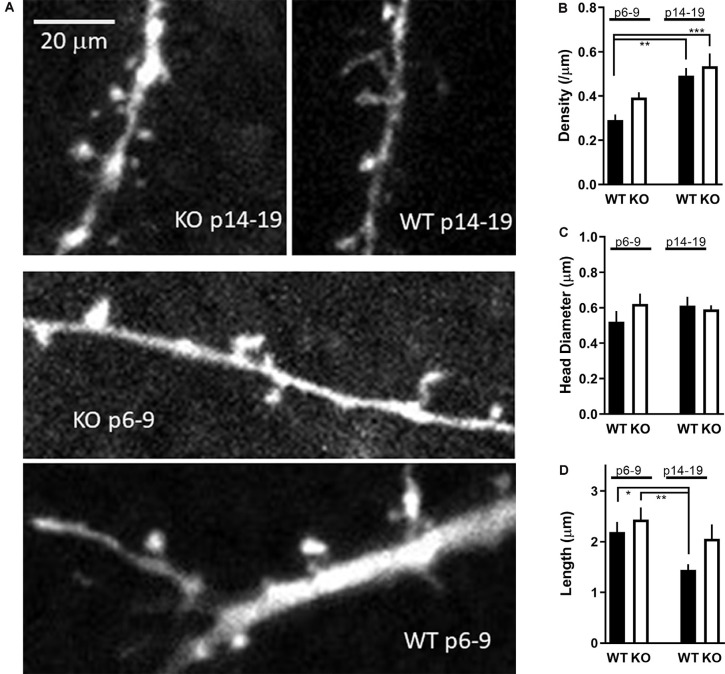
Development of dendritic spines in FMR1 KO CA1 neurons. **(A)** Sample images of apical dendrites with spines from WT and FMRP KO neurons as indicated in the figure. A total of 17–21 KO and 23–27 WT neurons were analyzed for spine morphology in the P6–9 and P14–19 groups, respectively. The scale bar is shown in the upper left is 20 μm. **(B)** Spine density in WT CA1 neurons (black bars) or FMRP KO neurons (white bars) from slices P6–9 or P14–19 as indicated. The asterisk indicates significance using one-way ANOVA (*F*_(1.959,15.67)_ = 8.663) with Tukey *post hoc* analysis. **(C)** Spine head diameter from CA1 neurons as in panel **(B)**. **(D)** Spine length from CA1 neurons as in panel **(B)**. Asterisk indicates statistical significance (one-way ANOVA *F*_(F1.872,14.35)_ = 6.831).

Spine density in WT neurons increased from 0.29 ± 0.03 to 0.49 ± 0.03 spines per micron (*p* < 0.01; Figure 7) similar to what has been previously reported in hippocampal pyramidal neurons (Gambrill and Barria, 2011). Similarly, spine density in KO neurons increased from 0.39 ± 0.02 to 0.53 ± 0.06 spines per micron (*p* < 0.05).

When spine density from P6–9 KO neurons is compared to P6–9 WT neurons, a slightly larger spine density in KO neurons is observed, consistent with the increase in mEPSCs frequency observed at this age (*p* < 0.01, *t*-test). Spine density between WT and KO neurons was no different at P14–19 (*p* > 0.5, *t*-test).

No significant difference was observed in the spine head diameter within the four groups (Figure 7C; *F*_(3,38)_ = 0.6272; *p* > 0.5, one-way ANOVA). Spine length in WT neurons decreased in a significant manner from P6–9 to P14–19 (*p* < 0.05; *t*-test) as expected as the spines are maturing. In contrast, spines in KO neurons remain at the same length (*p* > 0.5; *t*-test; Figure 7D), suggesting they are unable to properly mature perhaps due to the lack of LTP as observed earlier.

## Discussion

Both ASD and FXS are considered neurodevelopmental synaptopathies with an imbalance in excitatory and inhibitory neurotransmission (Spooren et al., 2012; Uzunova et al., 2014; Bagni and Zukin, 2019). The mechanisms that underlie learning and memory, cognitive, and social deficits associated with ASD and FXS are complex and depend on multiple factors including glutamate neurotransmission. ASD has a strong genetic component and genetic studies have implicated hundreds of genes associated with increased risk of ASD (Persico and Napolioni, 2013) where several members of the glutamate receptor family are included (Spooren et al., 2012).

Here, we have investigated the developmental profile of hippocampal CA1 principal neurons at two different developmental time windows; at P6–9 right when the first synaptic connection is being established in these cells, and P14–19, a period characterized by strong synaptogenesis and synaptic plasticity (Dailey and Smith, 1996; De Simoni et al., 2003; Gambrill and Barria, 2011).

Our electrophysiological recordings from FMRP KO slices focusing on ionotropic AMPA glutamate receptors have revealed a critical period by the end of the first 9 days after birth where the GluA2 subunit is upregulated. This confers synaptic AMPARs Ca^2+^ impermeability and a more linear I/V relationship at an age wherein WT slices AMPARs are more Ca^2+^ permeable and therefore could contribute to synaptic plasticity necessary for proper stabilization of nascent synapses (Park et al., 2018). We suggest that the improper brain development that takes place in KO mice is, at least in part, due to this abnormal expression of this important AMPAR subunit. Thus, it is well known that defects in GluA2 cause neurodevelopmental disorders. For example, Salpietro et al. (2019) found 28 *de novo* GluA2 mutations in unrelated patients with intellectual disability and neurodevelopmental abnormalities including ASD, Rett syndrome-like features, and seizures or developmental epileptic encephalopathy. Some of these mutations were found to be in the “Q/R” site affecting AMPAR calcium permeability, thus an alternative explanation could be dysregulation of ADAR2 (the Q/R editing enzyme) rather than a change in the expression of GluA2 protein itself.

The basis for the FXS phenotype is the lack of FMRP. The FMRP protein is highly localized to dendrites and spines (Pimentel, 1999; Bagni and Oostra, 2013) and has a wide variety of targets it is a selective RNA-binding protein that associates with polyribosomes and acts as a negative regulator of translation. FMRP has been shown to selectively bind approximately 4% of the mRNA in the mammalian brain (Ashley et al., 1993; Brown et al., 2001) and thereby affecting a wide variety of proteins, including many synaptic proteins and proteins involved in spine formation (Liu-Yesucevitz et al., 2011) and synaptic plasticity (Sidorov et al., 2013), including glutamate receptors (Sidorov et al., 2013). Expression and regulation of individual subunits that compose AMPARs, as well as the Ca^2+^ permeable NMDA-type glutamate receptors, happens in a very tightly orchestrated manner (Lohmann and Kessels, 2014). Any divergence from this can have serious consequences, including neurological conditions. Our results indicate a larger AMPAR to NMDAR ratio before postnatal 14 in slices from KO animals. This increase becomes normal after the beginning of the second postnatal week. This transient increase in AMPAR transmission could have developmental consequences in the formation of neuronal circuits, such that later, even though the ratio becomes normal, circuits have already been altered. The shift in the AMPAR/NMDAR ratio will also be influenced by impaired regulation of NMDARs known in the Fmr1 mouse. Disrupted NMDAR dependent synaptic plasticity has been reported mainly in the dentate gyrus (Yun and Trommer, 2011; Eadie et al., 2012; Franklin et al., 2014; Bostrom et al., 2015, 2016) but also in the CA1 area of the hippocampus (Toft et al., 2016; Lundbye et al., 2018). In contrast to our findings, Pilpel and colleagues reported a decreased AMPAR to the NMDAR ratio in the P14–16 age group in CA1 pyramidal neurons. The exact reason for this is unknown but it is important to point out that (Pilpel et al., 2009) used a double KO (Fmr1 KO2) and perhaps that remaining Fmr1 mRNA in the “conventional” Fmr1KO mouse (used here) are involved in synaptic regulation.

### Spine Morphogenesis

While the literature on dendritic spines in Fmr1 KO mice has been contradictory, there is a growing consensus that dendritic spine density is increased in Fmr1 KO hippocampus and cortex (Levenga et al., 2011; Pop et al., 2014; Jawaid et al., 2016; see also Bostrom et al., 2016 for review). Discrepancies in CA1 dendritic spine density and morphology in the KO are likely due to several variables including the age of the animals and the method of spine detection.

Interestingly, GluA2 has been reported to be directly involved in spine morphogenesis. Overexpression of GluA2 increases spine length, spine head width, and density in hippocampal cell cultures (Passafaro et al., 2003; Saglietti et al., 2007), all hallmarks of FXS spine abnormalities (He and Portera-Cailliau, 2013). Our data indicate that the upregulation of GluA2 happens during a critical developmental phase with intense synaptogenesis and synaptic pruning in the hippocampus and elsewhere in the brain. Interestingly, in the barrel cortex, a similar temporarily early development disruption of glutamate receptors was found as reported here (Harlow et al., 2010). Also in pluripotent stem cell lines generated from boys with FXS had altered GluA2 level compared to control (Achuta et al., 2018). Overall, our data combined with (Harlow et al., 2010), points toward a scenario where during early development, glutamate receptors are temporarily dysregulated. The temporary window might be short (just a few days) where after the receptors seems to settle down to normal. Thus, it is perhaps not surprising that the premature expression of this important synaptic protein influences the function and development of new synapses in the KO animal. Despite no clear difference in gross morphology in CA1 neurons from KO animals, we did found less branching for both apical and basal dendrites as determined by the number of dendritic crossing each concentric circle in Sholl analysis (Figure 5) as well as shifted first branch point in the KO (Figure 6). This is in agreement with previous reports that described similar findings in CA1 pyramidal neurons isolated from patients with autism (Raymond et al., 1996). Altered branching will ultimately lead to altered dendritic inputs and subsequent altered dendritic integration of inputs. On the other hand, dendritic spines density was upregulated in the immature KO age group to the same level as detected in the older age group. However, the only group where we could detect a maturing of spines was in the (P14–19) WT group where the average spine length shrunk over time suggesting more “mushroom” like spines than in the KO groups. In summary, at around the end of first postnatal week, an abnormal neuronal dendritic branching pattern is observed in CA1 pyramidal neurons from KO animals along with dendritic spines with a more immature morphology.

Altered synaptic glutamate receptors in the immature KO could interfere with the ability of the synapse to undergo plasticity. Early reports indicate that in mice lacking GluA2, LTP could still be induced (Jia et al., 1996). Since GluA2-lacking receptors are Ca^2+^ permeable (Burnashev et al., 1992), this suggested that CP-AMPARs may be alternative triggers for LTP at CA1 synapses. If the Ca^2+^ contribution of GluA2-lacking AMPARs is important for LTP early in development, it is reasonable to think that overexpression of GluA2 could diminish or eliminate LTP depending on the strength of the inducing protocol used. Here, we show that in the P6–9 KO group LTP was not observed suggesting that lack of Ca^2+^ permeable AMPARs limits the ability of synapses to undergo potentiation.

A wealth of information exists about LTP in the CA1 area in the KO with varying results from reduced (Lauterborn et al., 2007; Hu et al., 2008; Shang et al., 2009; Lundbye et al., 2018) to no difference (Godfraind et al., 1996; Li et al., 2002; Larson et al., 2005) in KO LTP. The vast majority of these LTP experiments are done in older mice than used here and thus the diversity of these results might be a result of recordings at different ages in addition to using different LTP-inducing protocols or other technical differences.

We observed a decrease in the frequency of AMPAR-mediated mEPSCs early in KO development as well as a decrease in their amplitude within the range of previous publications (Pfeiffer and Huber, 2007; Pilpel et al., 2009; Anggono et al., 2011; Luchkina et al., 2014; Lee et al., 2018). In addition to the lack of Ca^2+^ permeability, a larger fraction of synaptic AMPARs as indicated by larger mEPSCs amplitude could also contribute to occlude induction of LTP (Lledo et al., 1995).

The fact that synapses seem to be already potentiated with a larger ratio of AMPARs to NMDARs but morphological immature, suggests that synapses have not undergone regular plasticity. The increase in synapses observed, measured as an increase in spine density and increased mEPSC frequency with no changes in the pre-synaptic release, could be a compensatory cellular mechanism due to synapses that have not undergone required plasticity that stabilizes and selects some spines and synapses and allows removal of others.

What comes first: abnormal dendritic spine morphology, dysregulated synaptic proteins, or dysregulated synaptic plasticity? To solve the order of problems is challenging. What seems clear here, however, is that once the KO P6–9 reaches the P14 state, almost every parameter investigated here has gone back to normal and no differences with WT CA1 neurons are observed. In other words, the CA1 pyramidal neurons seem to have adapted to their changing environment. All the alterations observed early in development, increased AMPAR to NMDAR ratio, increased synaptic expression of GluA2, increased mEPSCs frequency and amplitude, immature spines, and lack of LTP, happen at a time when the brain is rapidly developing and any of these alterations will impact the developing neuronal circuits needed for proper brain function.

## Conclusion

One puzzling hallmark of the FMRP KO mouse is that yet, despite lacking the Fmr1 protein, despite having spine morphology issues, despites having plasticity issues, the KO phenotype is still roughly identical to WT, i.e., it eats and breed normally, and are by large hard to distingue from WT. This suggests that the mouse during early development seems able to adapt to it inherited “errors.” Here, we have established a critical period early in the Fmr1 KO mouse’ life where an important synaptic protein is dysregulated. As also demonstrated here this receptor is heavily involved in different types of synaptic plasticity and is directly involved in dendritic spine morphology. We found that about 2 weeks postnatal (5 days after the critical period), most of the abnormalities have normalized. This critical adaption period might explain why the Fmr1 KO mouse behaves largely as its WT counterpart.

## Data Availability Statement

The original contributions presented in the study are included in the article, further inquiries can be directed to the corresponding author.

## Ethics Statement

The animal study was reviewed and approved by Institutional Animal Care and Use Committee (IACUC) at the University of Washington.

## Author Contributions

TB and AB designed the experiments, analyzed the data, and wrote the article. All authors contributed to the article and approved the submitted version.

## Conflict of Interest

The authors declare that the research was conducted in the absence of any commercial or financial relationships that could be construed as a potential conflict of interest.
